# SquamataBase: a natural history database and R package for comparative biology of snake feeding habits

**DOI:** 10.3897/BDJ.8.e49943

**Published:** 2020-03-27

**Authors:** Michael C. Grundler

**Affiliations:** 1 Department of Ecology & Evolutionary Biology and Museum of Zoology, University of Michigan, Ann Arbor, United States of America Department of Ecology & Evolutionary Biology and Museum of Zoology, University of Michigan Ann Arbor United States of America

**Keywords:** Natural history, database, diet, snakes, squamate reptiles, macroevolution, macroecology

## Abstract

Public databases in taxonomy, phylogenetics and geographic and fossil occurrence records are key research tools that provide raw materials, on which broad-scale analyses and synthesis in their respective fields are based. Comparable repositories for natural history observations are rare. Publicly available natural history data on traits like diet, habitat and reproduction are scattered across an extensive primary literature and remain relatively inaccessible to researchers interested in using these data for broad-scale analyses in macroecology and macroevolution. In this paper, I introduce SquamataBase, an open-source R package and database of predator-prey records involving the world’s snakes. SquamataBase facilitates the discovery of natural history observations for use in comparative analyses and synthesis and, in its current form, contains observations of at least 18,304 predator individuals comprising 1,227 snake species and at least 58,633 prey items comprising 3,231 prey taxa. To facilitate integration with comparative analysis workflows, the data are distributed inside an R package, which also provides basic functionality for common data manipulation and filtering operations. Moving forward, the continued development of public natural history databases and their integration with existing digitisation efforts in biodiversity science should become a priority.

## Introduction

Understanding how organisms interact with their environment lies at the heart of evolutionary biology and ecology. The data that furnish this understanding come from the practice of natural history. Careful observations about diet, habitat, reproduction, behaviour and a range of other ecological traits are vital, not only for a basic understanding of an organism's way of life, but also for a broad array of more general questions in evolutionary biology and ecology. This is not a new perspective (e.g. Greene 2005). Natural history is fundamental to our understanding of a broad variety of phenomena, from diversity gradients to adaptive radiation to community assembly (Futumya 1998, Stroud and Losos 2016). Yet despite this central role for natural history, the growth of online public repositories for natural history data lags far behind comparable repositories for other types of data, such as nucleotide sequences and geographic occurrence records. Whereas large, specimen-based databases are available for these latter data types, they are woefully lacking for natural history observations (but see databases in Toledo et al. 2007; Schalk and Cove 2018; and Jobe et al. 2019).

This is surprising owing to the fact that such observations ultimately furnish the raw data used to test and challenge theoretical predictions (Greene 1986). Unexpected or unusual natural history observations, often dismissed out of hand as “anecdotal”, can also reveal novel patterns and spur new lines of enquiry when carefully catalogued (Boero 2013). For example, researchers who analysed thousands of anecdotal reports of unusual feeding behaviour in birds discovered that a clade’s rate of behavioural innovation is positively correlated with the ability of species to expand their geographic range (Sol et al. 2002), as well as with a clade’s species richness (Nicolakakis et al. 2003), lending support to the hypothesis that behavioural flexibility can drive accelerated rates of evolution and, more generally, to the idea that evolvability is an important driver of macroevolutionary patterns.

At a more fundamental level, publicly recorded natural history is essential for revealing the extent of our knowledge about the lives of other organisms. The widespread availability of field guides, carrying concise species accounts, can lead to the perception that much of the autecology of organisms is already known. This assumption is probably premature for the majority of life on Earth and our ability to identify knowledge gaps rests on the availability of natural history data (Hortal et al. 2015, Poisot et al. 2016). For example, after reviewing species accounts in a major compendium of mammal biology, only 38% of terrestrial mammals had recorded diet preferences (Kissling et al. 2014). The situation is undoubtedly worse for less charismatic groups of organisms.

Recognising the importance of and the need for repositories of publicly recorded natural history, standards-based frameworks capable of aggregating natural history observations from diverse sources are beginning to emerge (Poelen et al. 2014). Ideally, such initiatives will help identify and fill shortfalls in our knowledge of biodiversity and facilitate the discovery of natural history observations for use in comparative analyses and synthesis. In practice, the limited number of providers that maintain high-resolution natural history datasets make the realisation of these goals difficult.

Existing natural history datasets are generally derived from coarse summaries of the primary or secondary literature. For example, recent studies have used species accounts in major compendiums of bird and mammal biology to assemble global-scale datasets on traits like diet and foraging mode and these data have been used to address a range of questions in macroecology and macroevolution (Kissling et al. 2014, Pigot et al. 2016, Price et al. 2012, Wilman et al. 2014). However, the coarseness of such datasets can mask patterns that are apparent at finer scales (e.g. Borries et al. 2013), potentially limiting our ability to identify knowledge gaps and to develop novel lines of inquiry and analysis into how patterns of intraspecific trait variability are related to patterns at broader interspecific scales.

Natural history observations, like geographic occurrences or nucleotide sequences, are inherently tied to individual organisms, but unlike these latter, data can seldom be queried and downloaded at a specimen-based level. In the sections below, I briefly introduce and describe SquamataBase, an open-source R package and specimen-based database of predator-prey observations involving the world’s snakes.

## Installation

The development version of SquamataBase is hosted on Github and can be installed with the aid of the package devtools from within R, using the command devtools::install_github("blueraleigh/squamatabase"). The source code and commit history of the project can be viewed at:

https://github.com/blueraleigh/squamatabase.

Each stable release (including data and code) is also automatically archived with the Zenodo data repository. The current stable version is archived at:

https://doi.org/10.5281/zenodo.3667777

## Data Model

The core of SquamataBase is a database for storing data on specimens and trophic interactions between specimens. In the context of the SquamataBase data model, a "specimen" is a set of individual organisms (or components thereof) belonging to the same taxon (e.g. species, genus, family etc.). Each set has two measures of size (count and mass) and can be fleshed out with additional attributes if they are available, such as age, sex and body length. This generalised definition of a specimen to include multiple individuals is necessary because many publications present aggregate observations (e.g. 12 *Thamnophis sirtalis* ate 34 *Anaxyrus americanus* tadpoles), lacking individual-specific data. A generalised definition allows us to easily incorporate these observations alongside more specific observations.

A predator-prey interaction, or “food record" in SquamataBase terminology, is an observation of a snake specimen eating or attempting to eat a prey specimen. SquamataBase does not impose any particular categorisation of prey specimens, instead it simply records their taxonomic identities as stated by the original authors. Categorisation of prey specimens into a smaller number of groups for analysis is left to users (see below) because, in general, there will be many possible ways to categorise the original prey specimen taxonomic identities into a smaller number of prey types.

Each food record is linked to a reference publication where the data originate. Numerous contextual details are associated with a food record, including the basis for the record, whether the interaction was directly observed or inferred from evidence, the spatiotemporal context of the interaction, its outcome and details regarding habitat, ingestion direction and foraging strategy.

**Figure 1.**
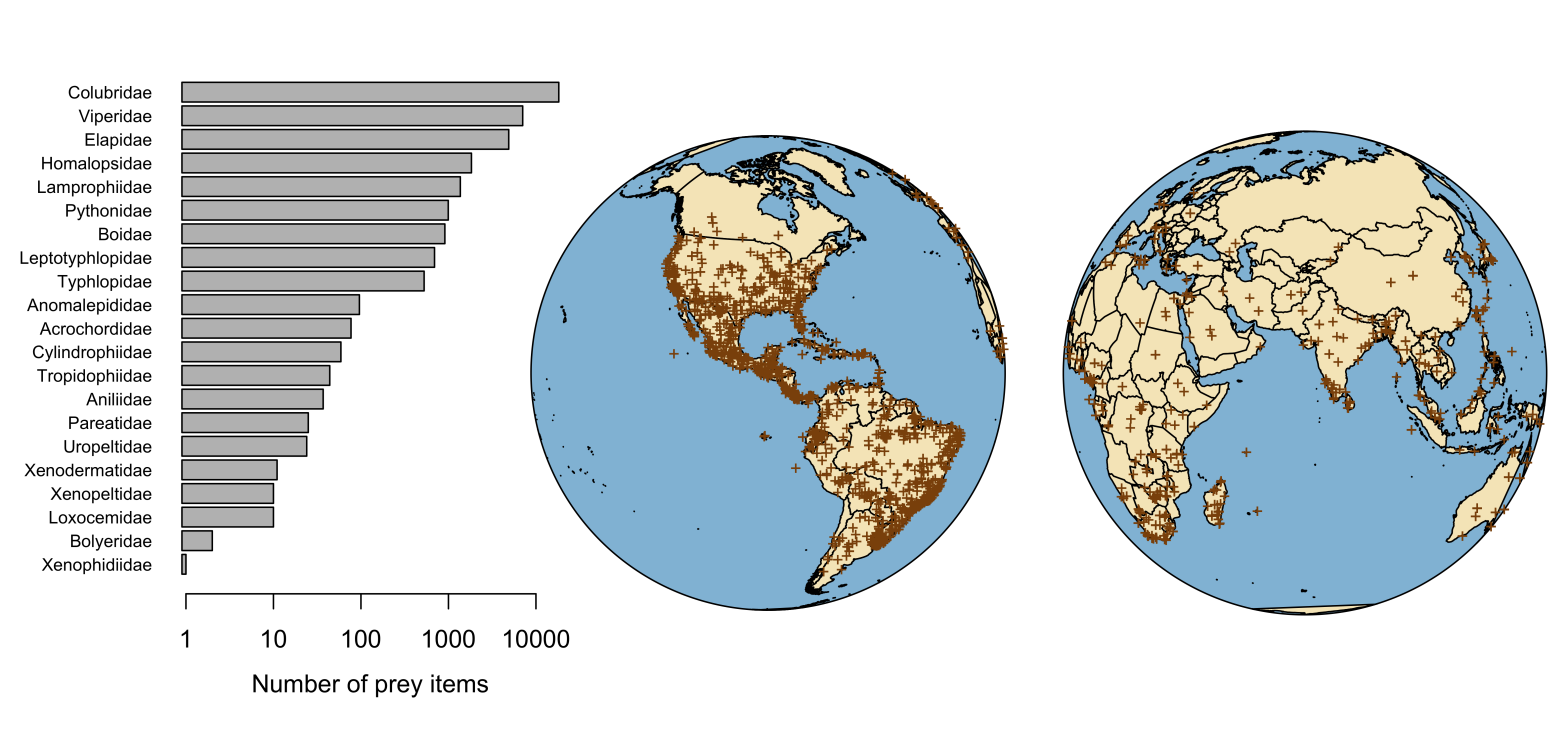
Predator-prey observations recorded in SquamataBase originate from a broad sampling of geographic regions and phylogenetic lineages. The bar graph illustrates the number of prey items currently recorded from major snake families. Many of these observations are georeferenced and their locations are illustrated as marks on the orthographic projections.

To ensure standardisation, all taxonomic names reported in reference publications are matched against the taxonomy provided from the Catalogue of Life (Roskov et al. 2016). Detailed documentation about each of the database fields, as well as the methods used to compile the data, are available in the package help documentation and can be accessed with the command help(diet). In its current form, the database contains observations of at least 18,304 predator individuals comprising 1,227 snake species and at least 58,633 prey items comprising 3,231 prey taxa. These observations originate from a broad sample of geographic regions and phylogenetic lineages (Fig. 1).

Approximately 1,700 different scientific publications currently serve as the source of observations recorded in SquamataBase. Relevant publications were located through the use of keyword queries in academic search engines and by a systematic review of table of contents for well-known herpetological journals (e.g. Herpetological Review, Herpetology Notes). I also located additional relevant articles by consulting the references in reviewed articles. Every effort was made to ensure that the same observation, reported in two different publications, was not also duplicated in SquamataBase (e.g. Gaiarsa et al. 2013 and Ferreto Fiorillo et al. 2013 report on the same specimen of *Mussurana bicolor* preying on a watersnake). The majority of observations in the database result from papers describing (1) dissections of fluid preserved museum specimens and (2) direct encounters with snakes in the field that were actively consuming a prey or had recently consumed a prey item that could be regurgitated by forced palpation. Glaudas et al. 2017 have noted that these sources of information can provide different pictures of the prey spectrum for *Bitis arietans* (Puff Adder).

## Filtering Records

SquamataBase provides functionality for filtering records by taxonomy and geography via the filter_records function. Taxonomic filtering can be performed on both predator and prey. For example, filtering records to only include observations from the snake genus *Chironius* is performed as:

> diet <- filter_records(predator_taxon = "Chironius")

To constrain this record further, we can pass the returned object to filter_records again with an additional criterion. For example, if we only wanted records involving prey items of the frog genus *Scinax* we would do:

> diet <- filter_records(diet, prey_taxon = "Scinax")

Geographic filtering can be performed with country level administrative names or with a bounding box. For example, the following line constrains the existing record set to only include records from Ecuador and Peru:

> diet <- filter_records(diet, locality_adm0_name = c("Ecuador", "Peru"))

Whereas the next line will constrain the existing record set to only include records lying between 80°W longitude and 60°W longitude and between 10°S latitude and the equator:

> diet <- filter_records(diet, xmin = -80, xmax = -60, ymin = -10, ymax = 0)

## Prey Classification

There are many ways to categorise prey items into different groups, but a relatively common categorisation scheme is simply to use higher prey taxonomy. SquamataBase therefore provides two out-of-the-box categorisation schemes that can be used to group prey specimens into a relatively small number of prey types according to higher taxonomy. These two built-in schemes also serve as examples of how users may programmatically devise their own categorisation schemes using the taxonomic metadata associated with each data record. The function that performs prey categorisation is group_prey and we invoke it on a record set like so:

> diet <- group_prey(diet, grouping = "coarse")

If the argument "grouping" is a character mode, then it must be one of "coarse" or "detailed", which correspond to the two built-in categorisation schemes alluded to above. In either case, the function returns a modified record set that contains an additional field identifying the prey category into which each prey specimen has been placed.

The group_prey function also allows users to define their own prey categorisation scheme and pass it to the function through the grouping argument. In this case, the argument must be a named list of functions, each one of which must return either TRUE or FALSE. For each record in the record set, each function in the list is tried, in order, until a TRUE value is returned. The name of the first function that returns TRUE is then the name of the prey group applied to the record. Arguments to these functions are expected to be fields that are present in the record set to which the prey grouping is being applied. Users can study the two built-in examples by inspecting the function bodies for the commands prey_coarse and prey_detailed.

## Aggregating Records

SquamataBase provides several options for aggregating records to create higher level summaries of the recorded prey items for snakes in a record set. These are available through the aggregate_records function. By default, the function will create a 3-column data frame with each row comprising a tuple of the form (*q, r, n*), where *q* is a snake species, *r* is a prey group and *n* is the number of recorded instances of *r* appearing in the diet of *q.* The optional "by" argument to the aggregate_records function serves to disaggregate this default layout by specifying a set of additional fields to preserve as columns in the result. For example, invoking the command aggregate_records(diet, by = "locality_adm0_name") will return tuples of the form (*q, r, p, n*) and *n* is now the number of recorded instances of *r* appearing in the diet of *q* in country *p.* Due to the nature of the data, there are several ways the value for *n* can be computed, because each data record contains the number *npred* of predator and the number *nprey* of prey individuals involved in the trophic interaction. The default behaviour of the function computes *n* by taking *min(npred, nprey)*, but this can be changed by the user through the use of function arguments.

## Conclusion

Shortfalls in our knowledge of species interactions and species trait distributions pose significant challenges to the study and understanding of biodiversity (Hortal et al. 2015). Specimen-based natural history databases can help delimit knowledge gaps and provoke solutions for their resolution (Poisot et al. 2016). By developing SquamataBase, my goal is simultaneously to facilitate the discovery and reuse of natural history data in comparative analyses and to encourage researchers to continue to publish and make available their observations. There is considerable scope for expanding the development of specimen-based natural history databases and integrating them with existing digitisation initiatives in biodiversity informatics and I suggest that this is a promising area in which to invest more effort.
